# Validation and reproducibility of the International Study of Asthma and Allergies in Childhood (ISAAC) Written Atopic Eczema Questionnaire for telephone survey in children aged 6–7 years^☆^

**DOI:** 10.1016/j.abd.2021.03.010

**Published:** 2022-01-03

**Authors:** Elisa Fontenelle de Oliveira, Camila Penedo, Solange Oliveira Rodrigues Valle, Fábio Chigres Kuschnir

**Affiliations:** a Department of Pediatrics, Instituto Nacional de Saúde da Mulher, da Criança e do Adolescente Fernandes Figueira, Fundação Oswaldo Cruz, Rio de Janeiro, RJ, Brazil; b Programa de Pós-Graduação em Ciências Médicas, Universidade do Estado do Rio de Janeiro, Rio de Janeiro, RJ, Brazil; c Department of Immunology, Universidade Federal do Rio de Janeiro: Programa de Saúde da Criança da Secretaria Municipal de Saúde e Defesa Civil do Rio de Janeiro, Rio de Janeiro, RJ, Brazil; d Department of Pediatrics, Faculdade de Ciências Médicas, Universidade do Estado do Rio de Janeiro, Rio de Janeiro, RJ, Brazil

**Keywords:** Atopic eczema, Epidemiology, Pediatrics, Validation study

## Abstract

**Background:**

The prevalence of atopic eczema is unknown in many countries. The International Study of Asthma and Allergies in Childhood (ISAAC) is an epidemiological landmark in the study of allergic diseases.

**Objective:**

To validate and assess the reproducibility of the ISAAC Written Atopic Eczema Questionnaire (WAEQ) for children aged between 6 and 7 years by telephone contact.

**Methods:**

Observational study through interviews with guardians of children aged 6 to 7 years using the ISAAC atopic eczema module questionnaire in three different phases separated by 2 weeks: telephone interviews in the first and third contacts and in-person interviews under supervision in the second contact. Reproducibility was estimated using the Kappa index and validation using the sensitivity and specificity coefficients.

**Results:**

Data from 88 children (32 from the atopic eczema group) were analyzed. Reproducibility showed almost perfect agreement for the questions “Recurrent pruritic lesions” and “Lesions in typical locations” (Kappa between 0.81–0.82), while a substantial agreement was observed for all other indicators (Kappa variation between 0.66 and 0.78). The validation showed high specificity (≥ 80.4%) and sensitivity (≥ 87.5%) for all questions, except those related to chronicity and medical diagnosis (34.4% and 40.6%, respectively).

**Study limitations:**

Non-random selection, no sample size calculation, participants from a tertiary hospital and study period coincident with the Coronavirus pandemic.

**Conclusions:**

Our results showed that the ISAAC atopic eczema module questionnaire by telephone interviews has good reproducibility and high agreement with the clinical diagnosis of atopic eczema. It may be an appropriate alternative tool in epidemiological studies of childhood atopic eczema, especially in periods of social isolation.

## Introduction

Atopic eczema (AE) is a common chronic inflammatory dermatosis that mainly affects children. It is often associated with a personal and/or family history of atopy. Pruritus is the most important symptom, which can progress to complications such as excoriation, infections, and sleep disturbances.^1^, ^2^

The prevalence and severity of AE is unknown in many countries. The International Study of Asthma and Allergies in Childhood (ISAAC) constitutes an epidemiological landmark in the study of asthma and other allergic diseases, such as AE and allergic rhinitis, allowing international and regional comparisons of prevalence and risk factors associated with these conditions.^3^ However, the administration of the ISAAC Written Atopic Eczema Questionnaire (WAEQ) for children between 6 and 7 years of age depended on the responses of their guardians in the household, which may have impacted the final results with a low response rate. Data from different centers participating in the phase 3 ISAAC in Brazil provided an AE prevalence of 8.2% in children in this age group.^4^

In 2006, due to the importance of Chronic Non-Communicable Diseases (NCDs) in the Brazilian population, the Ministry of Health implemented the VIGITEL system. This system interviews probabilistic samples of individuals aged 18 years and older who have a telephone in their homes, using questionnaires that address risk or protective factors for NCDs.^5^, ^6^ Computer-assisted telephone interviews are conducted annually; however, it does not include data on AE.^7^ This tool was shown to be faster, less expensive and more practical than the traditional methods.^8^

In a study carried out in the city of Rio de Janeiro, Valle et al. validated the written ISAAC asthma questionnaire for children aged 6 to 7 years by telephone interviews, showing good agreement and reproducibility of this method when compared to the original one.^5^

The aim of this study was to validate and evaluate the reproducibility of the WAEQ in children aged between 6 and 7 years administered to guardians through telephone interviews.

## Methods

This was an observational study carried out in Rio de Janeiro, Brazil, in a health unit aimed at education, research, assistance, technological development, and training in womens, children and adolescents health. This tertiary hospital receives patients from different neighborhoods and cities. Approximately 60% of the assisted families are low-income, and this same percentage of mothers have only elementary school education.^9^ The city of Rio de Janeiro, with 6,718,903 inhabitants (97.3% with telephone coverage) and a population density of 5265.82 inhabitants/km^2^, is located in the southeastern region of Brazil.^10^

This was a convenience sample. A search of medical records was carried out, and a total of 100 children aged between 6 and 7 years, divided into two groups, who had fixed or mobile telephone lines at home, were recruited to participate in the study. They ought to be undergoing follow-up for at least six months and be scheduled for consultation at the clinics in advance, sequentially, on every day of the week. Patients who were diagnosed with AE according to the UK diagnostic criteria established by specialists in the Dermatology and Allergy and Immunology clinics comprised the “AE” group.^11^ The “control group” consisted of children without dermatoses followed at the General Pediatrics outpatient clinic.

**Figure 1:**
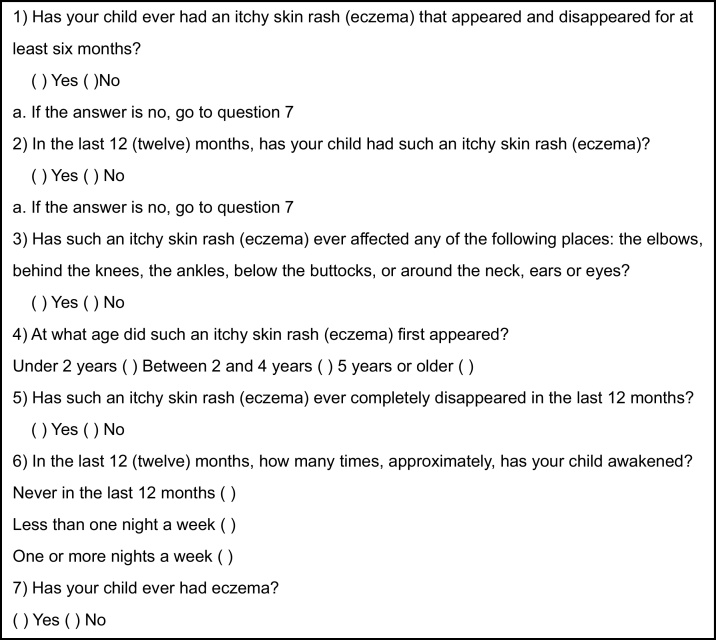
ISAAC Written Atopic Eczema questionnaire (WAEQ) for children between 6 and 7 years of age.
2 The second phase was carried out after two weeks, coinciding with the appointment date, so that the child’s guardian could fill in the WAEQ in person under the supervision of the main researcher and a physical examination could be performed to ascertain the absence of signs and symptoms of AE in the control group. On this occasion, the children from the AE group were submitted to the Scoring Atopic Dermatitis (SCORAD) analysis by one of the researchers. According to the score, the AE severity was considered mild up to 25, moderate between 25 and 50, and severe above 50 points.^13^
3 The third phase took place 15 days after the previous one when a researcher other than the first one conducted the second telephone interview using the WAEQ.

The study was carried out in 3 phases, in both groups:

1 The first phase consisted in applying the WAEQ (Fig. 1), previously validated for the Portuguese language, to the guardians of the children selected through telephone interviews.^12^ The main indicators assessed by this tool are the presence of current AE symptoms ("Recurrent pruritic lesions" and "Lesions in the last 12 months" - Questions 1 and 2) and typical locations of involvement ("Lesions in typical locations" - Question 3); “Age at onset” (under 2 years old, between 2 and 4 years old, or aged 5 and over – Question 4); severity of symptoms (“Complete improvement in the last 12 months” and “Sleep disturbances in the last 12 months” – Questions 5 and 6) and AE diagnosed by a physician (“Presence of eczema” – Question 7). According to the ISAAC protocol, the first question differentiates “typical” AE from other inflammatory dermatoses; the second focuses only on recent cases to minimize memory biases, such as incomplete data or selective memory; the third, which considers flexural involvement, associated with the fourth (“Age at onset”) substantially improved the specificity of the study; the fifth and sixth questions measure the dermatitis severity (chronicity and morbidity, respectively); and finally, the seventh may indicate a prior medical diagnosis.^3^ The questions were read slowly by one of the previously trained researchers, and the answers were recorded directly and immediately on a form and on an electronic record.

Only the data provided by the same guardians in all three stages of the study were considered for analysis of reproducibility and validation.

The reproducibility of questionnaires administered by telephone was calculated using the Kappa coefficient (κ). The results were classified as follows: above 0.81, almost perfect agreement; between 0.61 and 0.8, substantial agreement; between 0.41 and 0.6, moderate agreement; between 0.21 and 0.4, poor agreement; below 0.2, slight agreement.^14^

The validation was calculated by comparing the responses obtained during the first telephone interview with the UK clinical diagnostic criteria for AE used as the standard in our study.^11^ The specificity and sensitivity for each indicator and their respective accuracy were evaluated.

Subsequently, to assess the degree of agreement between the answers to the WAEQ filled out by the guardians on the day of the consultation with those obtained at the first telephone interview, the Kappa coefficient was used, with the same previously mentioned classification.

The data were stored in an Excel database, and statistical analysis was performed using SPSS software, version 23.

This study was approved by the Ethics Committee of the IFF. Written consent was replaced by verbal consent obtained prior to the interview, based on article N. 5 of Resolution N. 510, of April 7, 2016.

## Results

Between August 2019 and January 2020, 88 children were included in the study, 32 (36.4%) in the AE group and 56 (63.3%) in the control group. Fifty-two patients (59.1%) were male (64.3% in the control group and 50% in the AE group; p = 0.27). In the AE group, mothers accounted for 86.4% of respondents and fathers and grandparents, 6.25% each. In the control group, mothers, fathers and grandmothers were the respondents in 85%, 7.22%, and 7.22% respectively. There was no statistically significant difference regarding the percentage of this proportion between the two groups.

Due to the presence of more than one respondent for the same child in the different phases of the research or because the last interview was not carried out due to the new coronavirus pandemic, 4 patients (1 from the AE group and 3 from the control group) were excluded from the reproducibility assessment, and another 4 (1 from the control group and 3 from the AE group) were excluded from the agreement assessment process between the first telephone and the in-person interview. The average duration of each telephone interview was 5 minutes.

The distribution of severity according to SCORAD was 65.6%, 25% and 9.4% for mild, moderate, and severe AE, respectively. Patients in the control group were not submitted to this analysis because they did not have signs or symptoms of AE.

Table 1 shows the reproducibility of the WAEQ data obtained in the two telephone interviews. Almost perfect agreement was observed in the questions “Recurrent pruritic lesions” and “Lesions in typical locations”, while a substantial agreement was observed for all the other indicators.

**Table 1:** Reproducibility of atopic eczema indicators of the ISAAC written atopic eczema questionnaire (WAEQ) for children aged 6–7 years applied through telephone interviews with their caregivers (n = 84) Rio de Janeiro, 2020.

Indicator	Agreement n (%)^a^	Kappa Coefficient (κ)	95% CI	p-value
Recurrent pruritic lesions	76 (90.5)	0.82	0.71–0.93	<0.001
Lesions in the last 12 months	74 (87.9)	0.78	0.67–0.90	<0.001
Lesions in typical locations	76 (90.5)	0.81	0.69–0.93	<0.001
Age at onset	70 (83.4)	0.71	0.58–0.83	<0.001
Complete improvement in the last 12 months	68 (81.0)	0.66	0.52–0.79	<0.001
Sleep disturbance in the last 12 months	72 (85.7)	0.74	0.62–0.87	<0.001
Presence of eczema	77 (91.0)	0.70	0.52–0.96	<0.001

95% CI, 95% Confidence Interval.

a Total number and percentage of concordant responses between the 2 telephone interviews.

Table 2 shows the results of the validation of the WAEQ obtained through the first telephone interview with the guardians when compared with the clinical diagnosis given by a specialist based on the UK criteria. The first four questions (“Recurrent pruritic lesions”, “Lesions in the last 12 months”, “Lesions in typical locations” and “Age at onset”) and the sixth question (“Sleep disturbance in the last 12 months”) showed high sensitivity ( ≥87.5%) and specificity (≥80.4%). On the other hand, the fifth (“Complete improvement in the last 12 months”) and seventh (“Presence of eczema”) items showed low sensitivity (34.4% and 40.6%, respectively), but high specificity (91.1% and 98.2%, respectively).

**Table 2:** Validation of Written Atopic Eczema Questionnaire (WAEQ) eczema indicators for children aged 6–7 years obtained through telephone interviews with the guardians compared to the clinical diagnosis of atopic eczema (n = 88). Rio de Janeiro, 2020.

Indicator	Sensitivity (%)	Specificity (%)	Accuracy (%)
Recurrent pruritic lesions	93.8	80.4	85.0
Lesions in the last 12 months	87.5	91.1	89.0
Lesions in typical locations	87.5	96.4	93.0
Age at onset	87.5	91.1	89.0
Complete improvement in the last 12 months	34.4	91.1	70.0
Sleep disturbance in the last 12 months	87.5	91.1	89.0
Presence of eczema	40.6	98.2	77.0

When the two methods of administration (by phone and in-person) of the WAEQ were compared, discriminating the agreement of responses between them, there was an almost perfect agreement (κ ranging from 0.81 to 0.85) in the first three questions (“Recurrent pruritic lesions”, “Lesions in the last 12 months”, “Lesions in typical locations”), and substantial agreement (κ ranging from 0.71 to 0.76) in the last 4 indicators (Table 3).

**Table 3:** Agreement between the in-person responses to the Written Atopic Eczema Questionnaire (WAEQ) provided by caregivers of children aged 6–7 years and the responses obtained at the 1^st^ telephone interview (n = 84). Rio de Janeiro, 2020.

Indicator	Agreement n(%)^a^	Kappa Coefficient (κ)	95% CI	p-value
Recurrent pruritic lesions	78 (92.9)	0.85	0.73–0.96	<0.001
Lesions in the last 12 months	75 (89.3)	0.81	0.69–0.92	<0.001
Lesions in typical locations	77 (91.7)	0.83	0.70–0.95	<0.001
Age at onset	73 (86.9)	0.76	0.63–0.88	<0.001
Complete improvement in the last 12 months	71 (84.5)	0.71	0.57–0.84	<0.001
Sleep disturbance in the last 12 months	72 (85.7)	0.73	0.59–0.86	<0.001
Presence of eczema	79 (94.0)	0.76	0.56–0.96	<0.001

95% CI, 95% Confidence Interval.

a Total number and percentage of concordant responses between the 1^st^ telephone interview and the in-person interview.

## Discussion

The WAEQ applied by telephone interview showed good reproducibility in our study. The reproducibility analyzes the extent of inter- and intra-observer variability, which is often the main research question. The inter-observer variability describes the lack of reproducibility between two or more observers.^15^ Telephone interviews carried out with guardians of children aged between 6 and 7 years, by two different researchers, using the WAEQ demonstrated almost perfect or substantial agreement for all indicators of eczema, indicating that respondents understood the questionnaire, providing consistent responses to two observers, with a high level of standardization. A nearly perfect agreement was observed for "Lesions in typical locations", which is highlighted in the diagnostic criteria for AE, such as the Hanifin-Rajka and the United Kingdom criteria.^11^, ^16^

The ISAAC questionnaire aimed at adolescents aged between 13 and 14 years old, filled out directly by them at school, had a return rate higher than 95%; on the other hand, the return rate of the written questionnaire aimed at children between 6 and 7 years of age, which should be completed at home by their guardians, had only approximately 60% of return rate.^17^ In order to improve data quality and minimize losses in epidemiological studies, written questionnaires can sometimes be replaced by telephone interview, which is faster, less expensive, and more practical than the traditional methods.^6^

Studies using computer-assisted interviews have shown this technique to be useful in epidemiological surveys for chronic NCDs, such as cardiovascular diseases, in metropolitan areas of Brazilian states in the Southeast, South and Midwest regions.^6^ Additionally, such interviews have been successfully employed in these regions in studies focused on physical activity habits and sedentary lifestyles.^18^

In 2018, a study by the National Household Sample Survey (PNAD) carried out by the Brazilian Institute of Geography and Statistics (IBGE, *Instituto Brasileiro de Geografia e Estatística*) found that 9 of 10 Brazilian households had a mobile phone, 77.1% of people over 10 years old had mobile phones in 2016, being more than 80% in regions such as the Southern, Southeastern and Midwestern regions.^19^

Another significant sociodemographic aspect of our research is that, according to the Institute for Applied Economic Research (IPEA, *Instituto de Pesquisa Econômica Aplicada*), in 2015, around 45% of the families were supported by women.^20^ In addition, a study conducted in southeastern Brazil found that mothers of children in public schools are responsible for the feeding and hygiene of their children, being better able to answer questions about the health of their offspring.^21^ In our sample, more than 80% of respondents in both groups were mothers, suggesting that the information obtained about the presence or absence of eczema symptoms in their children was reliable.

Questionnaire validation was estimated by comparing the obtained responses with the clinical criteria used for the diagnosis of AE, showing the extent to which the correct answer was provided. The question “Lesions in typical locations” evaluated together with “Age at onset” are considered to have high sensitivity (proportion of subjects with AE whose answers were correct - true positives) and specificity (proportion of patients without AE who provided correct answers - true negatives).^15^, ^22^ High sensitivity and specificity were obtained for the first four questions, which contribute to the clinical diagnosis of recent AE. Moreover, the indicator “Lesions in typical locations” showed the best accuracy. The worst indicator, however, with an accuracy of 70%, was “Complete improvement in the last 12 months”, which indicates the chronicity of the disease.

The question “Presence of eczema” showed low sensitivity but high specificity. Probably because the term “eczema” (and not “dermatitis”) has limited use in our culture.^23^

The agreement between the first telephone interview and the in-person interview was almost perfect or substantial, indicating that these two different WAEQ methods had similar responses.

The present study has some limitations that can make it difficult to generalize its results, such as the non-random selection, absence of sample size calculation, restriction of the age group between 6 and 7 years, which may not be representative of other age groups, and exclusive participation of patients from a tertiary hospital. However, according to the SCORAD assessment, only 9.5% of the participants had severe AE, a percentage not very far from the mean prevalence of 6.0% of severe AE in Brazilian schoolchildren aged 6–7 years obtained by the phase 3 ISAAC.^4^

On the other hand, the clinical diagnosis of AE carried out by a specialist, the presence of only one respondent at all phases of the research and prior training of the researchers ensure good quality of data.

## Conclusion

Our results show that the WAEQ obtained through telephone interviews has good reproducibility and high agreement with the clinical diagnosis of AE made by a specialist, being able to discriminate children with and without the disease. Thus, it can be an appropriate alternative tool for epidemiological studies in AE, especially during a pandemic, such as the COVID-19, when social isolation is extremely important and in-person questionnaires become impossible to obtain.^24^

## Financial support

None declared.

## Authors’ contributions

Elisa Fontenelle de Oliveira: Design and planning of the study, collection, analysis, and interpretation of data; drafting and editing of the manuscript.

Camila Penedo: Design of the study and collection of data.

Solange Oliveira Rodrigues Valle: Design of the study and collection of data.

Fábio Chigres Kuschnir: Design of the study; analysis and interpretation of data; critical review of the manuscript.

## Conflicts of interest

None declared.
